# Prevalence of abnormal serum 25-hydroxyvitamin D and its association with hemoglobin level in pre dialysis CKD patients: a cross-sectional study from Himalayan country

**DOI:** 10.1186/s12882-019-1443-6

**Published:** 2019-07-17

**Authors:** Shiv Kumar Sah, Laxman Prasad Adhikary

**Affiliations:** 1 Department of Pharmacy, Little Buddha College of Health Science, Purbanchal University, Minbhawan, Kathmandu, Nepal; 2 Nephrology unit, Department of Medicine Kathmandu Medical Hospital Teaching Hospital, Sinamangal, Kathmandu, Nepal; 3 Little Buddha College of Health Science, GPO Box 26508, Minbhawan, Kathmandu, Nepal

**Keywords:** CKD, 25(OH) D, Hemoglobin

## Abstract

**Background:**

CKD has been recognized as risk factors for 25(OH) D deficiency, and Low levels of 25(OH) D have been suggested to be a trigger factor of decreased level of Hb. However, there is lack of information about the magnitude of 25(OH) D deficiency and Hb level in Nepalese CKD patients. Therefore, the aim of present study was to investigate the prevalence of abnormal 25(OH) D in non-dialyzed CKD patients, and further to examine its association with Hb level.

**Methods:**

In this cross-sectional study, we examined 172 clinically stable patients with an eGFR at CKD stage2–5 not on dialysis. Serum 25(OH) D, Hb, levels were evaluated as a core variables and the other variables such as age, sex, co-morbidities (HTN, DM), eGFR, Hb, iPTH, serum phosphate, albumin, calcium, and phosphate level were evaluated as a covariates. Serum 25(OH) D, Hb levels and the factors associated with 25(OH) D level were evaluated.

**Results:**

The estimated prevalence of abnormal 25(OH) D metabolite (< 30 ng/mL) in this predialysis patients were (87.8%), with 32 and 55.8% deficiency and insufficiency 25(OH) D metabolite, respectively. On regression analysis, serum 25(OH) D was positively associated with male subjects (*P* = 0.02), serum albumin(*P* = 0.002), and eGFR (*P* = 0.042), while inversely associated with age (*P* = 0.006), iPTH(*P* = 0.025). Hb concentration was found to be positively correlated with 25(OH) D (*P* < 0.05) in both univariate as well as in multivariate analysis.

**Conclusion:**

A high prevalence of abnormal 25(OH) D metabolite was observed in early CKD patients. Our study shows that lower level of 25(OH) D level are associated with lower level of Hb and higher level of iPTH, and could play a role in the development of anemia and hyperparathyroidism.

## Background

Chronic kidney disease (CKD) has been a growing health burden worldwide [1–4], and the patients with CKD continue to suffer from a wide range of complication including electrolyte imbalance, fluid overload, bone and mineral metabolism disorder to anemia [5, 6]. Anemia secondary to CKD is a complex complication which often goes untreated resulting in high morbidity, mortality, and cost of health care [7]. The incidence and prevalence of anemia increases as kidney function declines, and up to 50% of CKD patients not requiring chronic dialysis [7–10]. Although, erythropoietin deficiency, iron deficiency, and malnutrition–inflammation may have potential for the development CKD associated anemia, recent studies have indicated that 25(OH) D has pleiotropic effects on bone and mineral disorder [11, 12], and decreased level of 25(OH) D levels have been associated with low hemoglobin concentration in the subjects with normal renal function as well as in the patients with early chronic CKD [13–15]. Available literature suggests that high prevalent of 25(OH) D deficiency may be an important contributor to secondary hyperparathyroidism, which is harmful to bone health [16, 17]. Recently, epidemiologic studies have identified a high prevalence of suboptimal 25(OH) D levels in pre dialysis CKD patients [18, 19]. However, to date, such problem has not been extensively examined in Nepalese CKD population. Therefore, the present study was attempted to highlight the status of 25 (OH) D level in CKD patient not on dialysis, and further to examine whether the deficiency of 25(OH) D independently associated with the lower hemoglobin level.

## Methods

### Study design and duration

This cross-sectional study was carried out between June 2016 to May 2017 in the patients visiting outpatient nephrology unit at Kathmandu medical and teaching hospital.

### Study population and selection

A total 172 patients who met the inclusion criteria were included in the study. To be eligible for the study, individuals who were ≥ 18 years of age and have had measurement of serum creatinine and 25(OH) D and urine albumin creatinine. Clinical stable and with a diagnosis of CKD stage 2–5 who were not on dialysis were recruited for the study. The exclusion criteria included renal replacement therapy, a use of prescription based use 25(OH) D supplementation within 12 month of screening and phosphate binders.

### Variables of interest

The core variables of interest of this study was 25(OH) D level and hemoglobin concentration. The following covariates included in statistical analysis: age, sex, co-morbidities (HTN, DM), biochemical laboratory test including eGFR, Hb, iPTH, serum phosphate, albumin, calcium, and phosphate .

### Estimation of GFR

GFR were estimated by using MDRD-4 equation [20]:

$$ \mathrm{GFR}=175\mathrm{x}\ {\left(\mathrm{SCr}\right)}^{-1.154}\ \mathrm{x}\ {\left(\mathrm{age}\right)}^{-0.203}\ \mathrm{x}\ \left(0.742\ \mathrm{if}\ \mathrm{female}\right) $$

GFR units are mL/ min/1.73 m^2^. CKD staging was done as recommended by the National Kidney Foundation [21].

### Estimation of 25(OH) D and hemoglobin

Electrochemiluminescence immunoassay (ECLIA) test was used to measure the level of 25(OH) D and cyanmethemoglobin method for the estimation of Hb level .

Based on the opinion of most experts and available literature [15, 22], 25(OH) D was categories in three groups: vitamin D deficiency if serum 25(OH) D levels < 20 ng/mL, vitamin D insufficiency as 25(OH) D levels of 20–30 ng/mL, and vitamin D sufficiency as 25(OH)D3 levels ≥30 ng/mL. A cut-point less than 30 ng/mL of 25(OH) D was considered abnormal. All samples analyzed at a KMC-teaching hospital laboratory were utilized for the study.

### Data analysis

Continuous variables were expressed as mean ± SD or median ± interquartile range for skewed data. Based on 25(OH) D levels, the patients were categorized into three groups (deficiency: < 20 ng/mL, insufficiency: 20–30 and sufficiency: > 30 ng/mL). The significance of differences among continuous variables was performed by using One-way Analysis of Variance (ANOVA), and the significance of association for categorical measure was performed by Pearson’s Chi-square (휒2). Linear regression analysis were performed to examine the relationship between each 25(OH) D metabolite and clinical parameter including age, sex, calcium, phosphate, serum albumin, and serum iPTH. Multiple regression analysis was performed to assess the combined effects of clinical variables on serum level of each 25(OH) metabolite. Some missing data for iPTH, Ca and Phosphorous were imputed using multiple imputation regression method and were considered for analysis. A *p* value < 0.05 considered at level of significance for all statistical analysis.

### Ethical issues

Study protocol was approved by the institutional review board (IRB) of Kathmandu University and Teaching Hospital. Participants were well aware of the investigation and those who showed willingness to participate in the study voluntarily were consented prior to data collection.

## Results

Table 1 presents the prevalence of abnormal 25(OH)D. Total 172 patients were enrolled for the study. Prevalence of abnormal 25(OH) D (< 30 ng/mL) was 87.8% (95%; LL 82.2%-UL92.2%), with deficiency 32% (95%CI; LL25.1% -UL38.9%), and insufficiency (55.8%) (95%CI: LL-46.8%-UL-65.7%).

**Table 1:** Prevalence of abnormal 25(OH)D

25(OH)D	Patients n	Total (*n* = 172) Prevalence (95% CI)
Abnormal 25(OH)D	152	87.8% (82.2–92.2)
25(OH) D (strata)		
i. Deficiency(< 20 ng/mL)	55	32% (25.1–38.9)
ii. Insufficiency (20-30 ng/mL)	96	55.8% (46.8–65.7)
iii. Sufficiency (≥30 ng/mL)	21	12.2% (7.4–17.0)

Baseline characteristic of the enrolled subjects are presented in Table 2. Based on 25(OH) D levels, patients were categorized in three groups: group 1 (< 20 ng/mL), group 2 (20–30 ng/mL) and group 3(> 30 ng/mL). Compared with the group 1 and group3, mean ages were significantly higher in group 2 (< 0.05). Similarly, mean Hb levels were significantly different among the groups, and were found to be progressively increased as the 25(OH) levels increased (*P* < 0.05). There were no differences in sex, co -morbidities, SBP, DBP, Calcium, phosphorous, serum albumin, iPTH, eGFR and CKD stages amongst the group (*P* > 0.05).

**Table 2:** Characteristics of the patients according to serum 25(OH) D level

Variable	Total (*n* = 172)	Group1 < 20 *N* = 55	Group2 [20–30] *N* = 96	Group 3 > 30 *N* = 21	p
Age (years) ± SD	53.005 ± 15.33	52.75 ± 14.94	55.56 ± 14.75	42.09 ± 15.33	0.001
Sex, (%)					0.16
male	123 (71.5)	34 (19.76)	72 (41.86)	17 (9.88)	
Total (*n* = 172)	49 (28.48)	21 (12.20)	24 (13.95)	4 (2.32)	
Co-morbidities (%)					
HTN(+)	53 (30.81)	20 (11.62)	10 (5.81)	23 (13.37)	0.06
DM(+)	138 (80.23)	43 (25.00)	75 (43.60)	20 (11.62)	0.17
SBP ± SD	125.76 ± 15.51	126.96 ± 16.33	124.58 ± 15.17	127.86 ± 15.04	0.63
DBP ± SD	81.22 ± 10.94	81.42 ± 11.74	80.73 ± 11.00	82.84 ± 11.35	0.33
Hb (mg/dL) ± SD	12.23 ± 2.03	11.55 ± 1.90	12.48 ± 2.09	12.92 ± 1.84	0.006
Albumin (mg/dL) ± SD	4.05 ± 0.92	3.97 ± 0.37	4.08 ± 0.39	4.16 ± 0.49	0.10
Ca (mg/dL) ± SD	9.10 ± 1.19	8.96 ± 0.89	9.16 ± 1.41	9.19 ± 0.70	0.56
P (mg/dL) ± SD	3.59 ± 0.71	3.52 ± 0.56	3.65 ± 0.81	3.50 ± 0.60	0.48
iPTH (pg/mL) ± SD	235.96 ± 83.43	247.41 ± 60.25	235.51 ± 92.79	207.48 ± 51.32	0.10
SCr	2.66 ± 2.17	3.19 ± 2.91	2.36 ± 1.68	2.61 ± 1.66	0.081
eGFR (ml/min/1.72 m^2^)	46.14 ± 26.52	43.87 ± 25.59	46.04 ± 26.49	52.61 ± 29.28	0.82
CKD stage, n (%)					0.54
Stage 2 (eGFR> 60)	61	19 (11.04)	33 (19.18)	9 (5.23)	
Stage 3 (eGFR30–59)	51	19 (11.04)	28 (16.27)	4 (2.32)	
Stage 4 (eGFR 15–29)	37	8 (4.65)	23 (13.37)	6 (3.48)	
Stage 5 (eGFR < 15)	23	10 (5.81)	11 (6.39)	2 (1.16)	

Table 3 shows the factors associated with serum 25(OH)D. Age, sex, HTN, DM, eGFR, albumin, calcium, phosphorous and iPTH were modeled for the regression analysis. On univariate regression analysis, male subjects (β = .17,*P* = 0.02), serum albumin(β =0.23, *P* = 0.002), eGFR (β = 0.15, *P* = 0.042) positively and significantly linked to serum 25(OH) D level, while age (β = −.210, *P* = 0.006), and iPTH(β = −.17,0.025) were inversely and significantly associated.

**Table 3:** Association between 25(OH) D and clinical/biochemical parameter

Parameter	Univariate regression analysis		Multivariate regression analysis	
	β	P	β	P
Age (years)	−.210	0.006	−2.3	0.009
Sex (male)	.178	0.02	2.5	0.018
HTN(+)	.09	0.20		
DM(+)	.099	0.19		
eGFR (ml/min/1.72 m^2^)	0.15	0.042	0.005	0.95
Albumin (mg/dL)	0.23	0.002	0.20	0.007
Calcium (mg/dL)	0.068	0.37		
Phosphorous (mg/dL)	−.10	0.16		
iPTH (pg/mL)	−.17	0.025	−1.3	0.19

After adjusting age, sex, HTN, DM, serum albumin, EGFR, Ca, P, iPTH to the multiple regression analysis, only age (B = -2.3, *P* = 0.009), male (β =2.5,*P* = 0.018), serum albumin (β = − 0.20, *P* = 0.007) were found to be associated with 25(OH)D.

On univariate regression analysis, Hb concentration was found to be significantly correlated with age (β = − 0.025, *p* = 0.014), eGFR (β =0.49, *P* < 0.001), 25(OH) D (β = 0.31,*P* = < 0.001), iPTH (β = − 0.43, *P* < 0.001) and serum calcium level (β =0.28, *P* = < 0.001). The association between Hb concentration and serum 25(OH) D (β =0.14, *P* = 0.034) level, serum calcium (β =0.18, *P* = 0.004) and iPTH (β = −.20 *P* = 0.008) remained significant on multivariate regression analysis) (Table 4).

**Table 4:** Association between Hb and clinical/biochemical parameter

	Univariate regression analysis		Multivariate regression analysis	
	β	p	β	p
Age (years)	−.025	0.016	−0.25	0.80
Sex (male)	.67	0.052	0.10	0.094
HTN(+)	.043	0.581		
DM(+)	0.090	0.174		
eGFR (ml/min/1.72 m^2^)	0.49	< 0.001	0.27	< 0.001
25(OH) D (ng/mL)	0.31	< 0.001	0.15	0.022
Albumin (mg/dL)	0.32	< 0.001	0.17	0.009
Phosphorous (mg/dL)	−0.004	0.95		
Ca (mg/dL)	0.28	< 0.001	0.20	0.002
iPTH (pg/ml)	−0.433	< 0.001	−.203	0.008

**Fig. 1:**
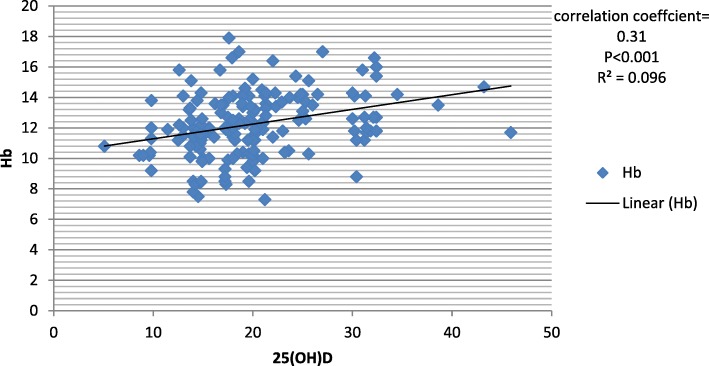
Relationship between 25(OH) D and Hb

Figure 1 depicts the correlation between 25(OH) D and Hb. Pearson correlation coefficient revealed a significant and positive correlation between the 25(OH) D and Hb (*r* = 0.31,*P* = < 0.001).

## Discussion

Recent studies have shown to be a high prevalence of 25(OH) D deficiency across the nations [23–25]. However, the assessment of 25(OH) D in CKD population has not been in routine practice in the resource limiting country, especially in Nepal. And also the magnitude of such problem has not been addressed properly. To the best of our knowledge this the first study that has been attempted to highlight the status of 25(OH) D and hemoglobin in patients with CKD stage 1–5 not on dialysis in Nepalese setting.

In the present study, we observed 87.8% population had abnormal 25(OH) D (< 30 ng/mL) level, with 32% deficiency and 55.8% insufficiency 25(OH) level. In agreement with this finding, most recently in US study, 86% had suboptimal levels of vitamin D (< 30 ng/ml) [25]. Similarly, previous study [14] in chronic kidney disease demonstrated that 3.11% had 25(OH) D (< 10 ng/mL) deficiency, 54.51% insufficiency (10-30 ng/mL). Likewise, in a earlier report [26], 57 and 58% population had 25(OH) D insufficiency (10-30 ng/mL) in CKD stage 3 and 4 respectively; and 14 and 26% had calcidiol deficiency(< 10 ng/ml) in CKD stage 3 and 4 respectively. These widespread variations in the prevalence of vit D deficiency may be explained by the number of factors including variation in the degree of renal impairment, co-morbidities and the diversity of the enrolled population in the respective studies.

The pathophysiology of 25(OH) D deficiency is multifactorial and is varied by race, sunlight exposure, and presence of risk factors such as age, type-2 diabetes and obesity, and other co-morbidities [13]. Serum levels of vitamin D metabolites are also affected by several factors including calcium, phosphate, iPTH, and the progression of CKD [23–25].

By linear regression analysis we observed a significant correlation between 25(OH) D and clinical/biochemical parameter. Age, sex, eGFR, serum albumin and iPTH were found to be significantly correlated with serum 25(OH)D. Further we applied multiple regression to determine the combined influence of these factors on serum level of 25(OH), and observed that age, sex and serum albumin was independently associated with serum 25(OH)D.

In the present study, we noted that age appeared to be inversely correlated with serum 25(OH) D and also remained associated in multiple regression analysis, which compiles the results with previous study [23]. Thus, the data imply that age is an important predictors for serum 25(OH) D, where increasing age is associated with the decreased level of serum 25(OH)D. Also we noted that high level of serum 25(OH) D level was associated with male gender, and this affirm the result of the previous study of early CKD patients [23].

By general linear regression, there was a significant positive correlation between 25(OH) D and eGFR. These results supports the observations from the large number of previous studies in which serum 25(OH) level decreased as the renal function declined early in the course of CKD [18, 19].

In this study, low level of serum albumin level was positively correlated with the low level of 25(OH) D level; indicating that hypoalbuminemia may be one of the potential contributing factor to the low level of 25(OH) D levels. It is widely accepted that 25(OH) D deficiency may lead to hyperparathyroidism in normal individuals [27, 28]. Similarly, the present study shows a significant, inverse relationship between 25(OH) D levels and iPTH, indicating that low level of 25(OH) D could influence the high level of iPTH,. This finding is consistent with a large number of studies in different populations and suggests that low level of 25(OH) D could lead to secondary hyperparathyroidism, which is harmful to bone health [16, 17]. Our study finding also support the concern raised by the K/DOQI guideline that the high prevalent of 25(OH) D deficiency may be an important contributor to the pathogenesis of secondary hyperparathyroidism, which has been largely overlooked in our study population. In the present study, low level of 25(OH) D was strongly correlated with reduced eGFR; suggesting that deterioration of renal function may be related to the deficiency of 25(OH)D. And the association is congruent with a large number of epidemiologic studies [14, 23, 25].

In the present study we also attempted to ascertain the association of Hb with serum 25(OH) D, and other biochemical parameter. The anemia secondary to kidney disease is complex and a number of factors attributes to this pathogenesis [29]. Recently, some studies have reported the positive relationship between serum 25(OH) D and Hb concentration in general population as well as with chronic kidney disease patients [13, 23]. Although, the exact pathophysiology of this association is unclear, it is believed that 25(OH) D deficiency could lead to increased risk of reticulocytosis and iron deficiency anemia [30]. In bone marrow there are enormous 25(OH) D receptors and the presence of high local concentration of 25(OH) D in hematopoietic tissue is suggested to activate erythroid precursors cell [31].

Our study demonstrated that Hb concentration significantly, positively correlated with 25(OH) D age, eGFR and calcium level. Conversely, there was inverse relationship between Hb and iPTH. Also, after controlling for the other important covariates, the effects of serum 25(OH) D level, serum albumin, calcium and iPTH remained significant predictors of hemoglobin. And this association is well described in some recent studies in early CKD population [14, 15].

### Limitations

Since, the nature of the study being cross-sectional, the study only allows evaluating the association but could not draw the any conclusion about causal relationship over the time. Thus, an interventional study in a large population is needed to explore the causal relationship between 25(OH) D and Hb.

## Conclusion

Abnormal 25(OH) D metabolite is commonly seen in Nepalese CKD patients not on dialysis, and that 25(OH) are directly correlated with male subjects, kidney function, and serum albumin, while inversely linked to age and iPTH. Also, lower level of 25(OH) D have shown to be associated with lower level Hb. The findings indicate that pre-dialysis CKD patients are at a greater risk of developing anemia and hyperparathyroidism, and a timely intervention and proper management of hypovitaminpsosis, especially in elder male population with advanced CKD pre-dialysis patients, is crucial so as to prevent from the disorder that may arise due to 25(OH) D insufficiency.

## Data Availability

The datasets of the current study will be made available from the corresponding author on reasonable request.

## Abbreviations

Ca: Calcium
CKD: Chronic kidney disease
DBP: Diastolic blood pressure
eGFR: Estimated glomerular filtration rate
Hb: Hemoglobin
iPTH: Intact paratahyroid hormone
P: Phosphorous
SBP: Systolic blood pressure
